# Estudo Comparativo da Doença Coronariana Microvascular Causada por Doença de Chagas e por Outras Etiologias

**DOI:** 10.36660/abc.20200381

**Published:** 2020-12-01

**Authors:** Felipe Araujo Campos, Mariana L. Magalhães, Henrique Turin Moreira, Rafael B. Pavão, Moyses O. Lima, Igor M. Lago, André V. Badran, João R. A. Chierice, André Schmidt, José Antonio Marin

**Affiliations:** 1 Universidade de São Paulo Faculdade de Medicina de Ribeirao Preto Ribeirão PretoSP Brasil Universidade de São Paulo Faculdade de Medicina de Ribeirao Preto, Ribeirão Preto, SP - Brasil

**Keywords:** Cardiomiopatia da doença de Chagas, Doença de Chagas/complicações, Disfunção Microvascular Coronariana, Disfunção Ventricular Esquerda, Escore de Anormalidade Sistólica Parietal, Fração de Ejeção Ventricular

## Abstract

**Fundamento:**

A doença de Chagas (DC) constitui uma causa potencial negligenciada de doença microvascular coronariana (DMC).

**Objetivos:**

Comparar pacientes com DMC relacionada à DC (DMC-DC) com pacientes com DMC ligada a outras etiologias (DMC-OE).

**Métodos:**

De 1292 pacientes estáveis, encaminhados para angiografia coronária invasiva para elucidar o padrão hemodinâmico e a causa de angina, 247 apresentaram coronárias subepicárdicas normais, e 101 foram incluídos após aplicação dos critérios de exclusão. Desses, 15 compuseram o grupo de DMC-DC e suas características clínicas, hemodinâmicas, angiográficas, e cintilográficas foram comparadas às do grupo de 86 pacientes com DMC-OE. O nível de significância estatística para todas as comparações adotado foi de 0,05.

**Resultados:**

Pacientes com suspeita de DMC-DC apresentaram características antropométricas, clínicas e angiográficas, além de alterações hemodinâmicas e de perfusão miocárdica estatisticamente comparáveis às detectadas nos 86 pacientes com DMC-OE. Disfunção ventricular diastólica, expressa por elevada pressão telediastólica do ventrículo esquerdo, foi igualmente encontrada nos dois grupos. Entretanto, em comparação a esse grupo com DMC-OE, o grupo com DMC-DC exibiu fração de ejeção ventricular esquerda mais baixa (61,1 ± 11,9 vs 54,8 ± 15,9; p= 0,049) e mais elevado escore de mobilidade da parede ventricular (1,77 ± 0,35 vs 1,18 ± 0,26; p= 0,02).

**Conclusão:**

A cardiomiopatia crônica da doença de Chagas esteve associada à etiologia de possível doença microvascular coronariana em 15% de amostra de 101 pacientes estáveis, cujo sintoma principal era angina requerendo elucidação por angiografia invasiva. Embora os grupos DMC-DC e DMC-OE apresentassem características clínicas, hemodinâmicas, e de perfusão miocárdica em comum, a disfunção global e segmentar do ventrículo esquerdo foi mais grave nos pacientes com DMC associada à DC em comparação à DMC por outras etiologias. (Arq Bras Cardiol. 2020; 115(6):1094-1101)

## Introdução

Mais de um século após sua descoberta em 1909, a doença de Chagas (DC) ainda é sério problema de saúde pública na América Latina e, devido a intensos movimentos migratórios nas últimas décadas, também em áreas não endêmicas, como nos Estados Unidos e em alguns países europeus.^1 , 2^ A cardiomiopatia crônica da doença de Chagas (CCDC) é a mais prevalente e mais grave das manifestações clínicas da DC, sendo causada, essencialmente, por miocardite infecciosa de baixo grau, mas praticamente incessante, difusa, com necrose miocitolítica focal e intensa fibrose reativa e reparadora.^3 , 4^ Em modelos experimentais de isquemia/reperfusão, a necrose miocitolítica é geralmente identificada como um processo de baixa intensidade, mas com danos hipóxicos ou isquêmicos iterativos. Por isso, tem sido interpretada como uma consequência de distúrbios isquêmicos microvasculares, constituindo um dos quatro mecanismos patogenéticos fundamentais da CCDC.^5 , 6^

A ocorrência de isquemia microvascular em pacientes com infecção crônica pelo *T. cruzi* foi demonstrada por várias evidências patológicas, clínicas e experimentais.^7 - 9^ Estima-se que 20-40% dos pacientes com CCC queixem-se de angina, geralmente de caráter atípico, uma vez que o sintoma não tem relação direta com esforço físico ou estímulo emocional, além de duração e resposta a nitratos variáveis.^10^ Além disso, vários pesquisadores evidenciaram que esses pacientes apresentam anormalidades marcantes na perfusão miocárdica provocadas pelo exercício e reversíveis com o repouso, em presença de artérias coronárias sem lesões epicárdicas obstrutivas na angiografia invasiva.^11 - 13^ Essas anormalidades na perfusão são, portanto, atribuíveis à isquemia microvascular, e são corroboradas por estudos em modelos experimentais de infecção pelo *T. cruzi.* ^14 , 15^

Com base nessas evidências, muitos pacientes com CCC poderiam ser classificados como portadores de uma classe secundária de doença microvascular coronariana (DMC), relacionada a uma cardiomiopatia causada por doença infecciosa inflamatória crônica.^16 - 18^

Não há estudos com foco na comparação de pacientes com DMC relacionada à DC com pacientes com DMC atribuída a outras etiologias. Dessa forma, esse foi o objetivo da presente investigação.

## Métodos

### Delineamento do Estudo e Amostra Populacional

Esta foi uma investigação transversal, observacional e unicêntrica, com inclusão prospectiva de pacientes estáveis, que foram encaminhados a nosso hospital universitário terciário entre 01 de janeiro e 31 de dezembro de 2018, para realização de angiografia coronariana invasiva, para elucidar o padrão hemodinâmico e a causa de *angina pectoris* , referida como principal sintoma na história clínica. De total de 1292 pacientes, 601 foram excluídos devido a: tratamento prévio com angioplastia coronariana (n = 200); síndrome coronariana aguda confirmada prévia (n = 137); cardiopatia valvar (n = 113); cardiomiopatia dilatada hipertrófica ou idiopática (n = 99); cirurgia prévia de revascularização do miocárdio (n = 49) e transplante cardíaco (n = 3).

Nos 691 pacientes restantes, a angiografia coronariana (indicada para avaliar a possibilidade de doença arterial coronariana) revelou: lesões epicárdicas obstrutivas significativas (estenoses> 40% de redução do diâmetro luminal) (n = 367); artérias coronárias angiograficamente normais (n = 247); doença arterial coronariana epicárdica não significativa - estenoses <40%: (n = 77); anomalias das artérias coronárias - anomalias congênitas, ponte miocárdica, tortuosidade excessiva ou ectasia, fístula coronário-cavitária, fluxo lento (n = 81).

Dos 247 pacientes clinicamente estáveis, sem doença cardíaca estrutural e cujo sintoma principal era angina (grave o suficiente para justificar a indicação de angiografia coronariana invasiva), sem anormalidades à angiografia, 101 concordaram em participar do estudo e assinaram o termo de consentimento. O protocolo de pesquisa foi aprovado pelo Comitê de Ética do Hospital das Clínicas da Faculdade de Medicina de Ribeirão Preto - USP - Processo: 8430/2011 e CAEE 07494618.3.0000.5440 Doc 3.252.539.

### Avaliação Clínica e Laboratorial

Os 101 pacientes incluídos no estudo passaram por avaliação clínica e hemodinâmica, além de angiografia coronariana invasiva no mesmo dia, para caracterização da angina, avaliação de sintomas associados tais como dispneia, fadiga, edema, palpitação, síncope, disfagia, constipação intestinal, e indícios epidemiológicos de exposição ao *T. cruzi* .

Também foi aplicado um questionário sobre fatores de risco para doença arterial coronariana, tais como: hipertensão arterial sistêmica, diabetes mellitus, dislipidemia, tabagismo atual e história familiar de doença coronariana precoce. Todos os pacientes realizaram um eletrocardiograma (ECG) de 12 derivações e um subgrupo de 33 (nove e 24 pacientes em cada grupo) realizou ecocardiograma transtorácico de repouso. Todos os 101 pacientes realizaram exame sorológico para detecção de anticorpos contra o *T. cruzi.* Além disso, uma amostra de sangue periférico foi coletada de todos os pacientes para determinação dos níveis séricos de creatinina, e exclusão de danos renais e hepáticos, bem como de anemia e diabetes mellitus.

### Cateterismo Cardíaco, Avaliação Hemodinâmica e Angiografia Coronariana

Os procedimentos de cateterismo cardíaco, avaliação hemodinâmica e angiografia coronariana foram realizados sob anestesia local, preferencialmente por abordagem radial, com fios-guia e cateteres convencionais. Injeções manuais de 3-7 mL de contraste radiológico foram feitas seletivamente em cada óstio coronário, com gravação de 15 a 30 quadros/s, em várias projeções.^19^ A pressão telediastólica do ventrículo esquerdo (PTDVE) foi registrada em repouso, seguida de duas injeções automáticas de 20-30 ml de contraste a 8-10 ml/s e gravação a 15 quadros/s, nas projeções oblíquas direita e esquerda. A ventriculografia de contraste foi então analisada de acordo com um modelo de 9 segmentos, utilizando um escore de quantificação que atribuiu 1 à mobilidade normal da parede, 2 à hipocinesia, 3 à acinesia e 4 à discinesia.^20^ Um escore médio de movimentação segmentar da parede ventricular foi obtido pela soma das pontuações de cada segmento, dividida pelo número de segmentos analisados^21^ conforme publicação recente de nossa instituição. O ventriculograma de contraste radiológico também permitiu a avaliação qualitativa de hipertrofia e dilatação do ventrículo esquerdo.

### Cintilografia Miocárdica de Perfusão com Método SPECT

Um subgrupo de 19 pacientes foi submetido à cintilografia miocárdica de perfusão por tomografia computadorizada por emissão de fóton único, em repouso e durante estresse físico ou farmacológico. As imagens foram obtidas por câmera (Philips BrightView XCT - Cleveland, OH) de detector duplo, com o paciente na posição supina. A aquisição ocorreu em órbita semicircular (180 graus, da projeção oblíqua anterior direita para a projeção oblíqua posterior esquerda), em 32 projeções sincronizadas com o eletrocardiograma, 8 quadros por ciclo cardíaco em 60 segundos por projeção com 50% de janela de aceitação em torno do RR médio. As imagens foram adquiridas com detectores equipados com colimadores de orifícios paralelos de baixa energia e alta resolução, utilizando uma matriz de aquisição de 64 x 64 pixels.^22^

O exercício físico foi utilizado preferencialmente como o teste de estresse. Betabloqueadores, bloqueadores de canais de cálcio e outros medicamentos anti-isquêmicos foram interrompidos 48 horas antes dos testes nucleares. O sestamibi-Tc^99m^ foi utilizado como radiotraçador para avaliar a perfusão miocárdica regional, na dose de 12 a 15 mCi em repouso e 25 a 30 mCi em estresse. As imagens foram obtidas 1 hora após cada injeção intravenosa do radiotraçador.

Mapas polares, usando o modelo de 17 segmentos, foram gerados para avaliar a anormalidade da perfusão de acordo com uma pontuação para cada segmento definida como 0 = normal, 1 = redução leve da captação, 2 = redução moderada da captação, 3 = redução acentuada da captação, e 4 = ausência de captação. As anormalidades de perfusão no estresse (SSS - *Summed Stress Score* ) e repouso (SRS - *Summed Rest Score* ) foram quantificadas para diferenciar os defeitos reversíveis – quando o *Summed Difference Score* (SDS) foi ≥ 1, de defeitos perfusionais irreversíveis.^23^

### Análise Estatística

Com base nos testes sorológicos específicos para detecção de anticorpos contra o *T. cruzi* , 15 pacientes foram classificados como portadores de provável DMC causada por doença de Chagas (DMC-DC) - dois testes sorológicos positivos com métodos diferentes.^24^ Os outros 86 pacientes, com testes sorológicos negativos, compuseram o grupo de pacientes com provável DMC por outras etiologias (DMC-OE). Os testes de Shapiro-Wilk foram utilizados para verificar se as variáveis tiveram uma distribuição normal; as variáveis não pareadas foram comparadas com teste t de *Student* ou o teste de Mann-Whitney não pareado. As variáveis contínuas com distribuição normal foram descritas como média ± desvio-padrão (DP), enquanto as variáveis com distribuição não normal foram descritas como mediana e intervalo interquartil (IQ). As variáveis categóricas foram descritas como valores absolutos ou relativos (percentuais ou proporções). As proporções dentro de cada grupo foram comparadas usando teste exato de Fisher. Todos os testes foram bicaudais, com p < 0,05 considerado como significante. Todas as análises foram feitas utilizando o programa Stata *(StataCorp, EUA, versão 14.2)* .

## Resultados

### Características clínicas - (Tabela 1)

Dos 247 pacientes estáveis que preencheram os critérios de inclusão e eram considerados elegíveis, 101 (40,9%) foram recrutados. Desses, apenas 15 (14,8%) apresentaram dois testes sorológicos positivos com anticorpos contra *T. cruzi* e compuseram o grupo DMC-DC (40% homens e idade média de 61,3 ± 6,7 anos). Os outros 86 (85,2%) compuseram o grupo DMC-OE (32,5% homens e idade média discretamente mais elevada, de 68,9 ± 11,0 anos).

Na data de início do estudo, a angina atípica foi referida por 9 (60%) pacientes do grupo DMC-DC versus 57 (66%) pacientes do grupo DMC-OE, enquanto os demais pacientes de ambos os grupos referiram presença de angina típica. No grupo DMC-DC, dispneia e palpitação também foram frequentes, com 8 (53%) e 7 (47%) pacientes, respectivamente, versus 55 (64%) com dispneia e apenas 30 (35%) com palpitações no grupo DMC-OE.

A hipertensão arterial sistêmica foi o fator de risco mais prevalente para doença arterial coronariana nos dois grupos, com 93,3% vs 81,3%, seguida de *diabetes mellitus* com 40,0% vs 33,7%, dislipidemia com 33,3 vs 41,8% e tabagismo ativo em 13,3 vs 24,4%, respectivamente nos grupos DMC-DC e DMC-OE. Ambos os grupos estavam usando proporções estatisticamente semelhantes de medicamentos para hipertensão, diabetes mellitus, e agentes usados no tratamento de isquemia miocárdica, como estatinas, antiagregantes plaquetários e antagonistas dos canais de cálcio ( Tabela 1 ). Entretanto, os inibidores da enzima de conversão de angiotensina e bloqueadores dos receptores de angiotensina II foram mais usados no grupo DMC-DC, enquanto os agentes antiplaquetários eram mais comuns nos pacientes do grupo DMC-OE.

**Tabela 1:** – Características demográficas e clínicas basais dos pacientes inseridos conforme a etiologia da doença microvascular coronária

	DMC-DC n = 15	DMC-OE n = 86	Valor p
Idade (anos)	61,3 ± 6,7	68,9 ± 11,0	0,01
Gênero feminino (%)	60,0	67,4	0,65
Peso corporal (kg)	77,0 ± 13,1	80,7 ± 15,3	0,18
IMC (kg/m^2^)	31,0 ± 7,3	31,9 ± 5,6	0,72
Angina atípica (%)	60,0	66,3	0,86
Dispneia (%)	53,0	64,0	0,62
Palpitação (%)	47,1	35,2	0,56
Hipertensão (%)	93,3	81,3	0,46
Diabetes mellitus (%)	40	33,7	0,77
Dislipidemia (%)	33	53,5	0,58
Tabagismo (%)	13,3	24,4	0,51
**Medicações em Uso**			
IECA/BRA	100	71	0,037
Betabloqueador	53	54	0,79
Estatinas	47	53	0,90
Antidiabéticos	40	42	0,88
Diuréticos	47	40	0,82
Nitratos	20	12	0,63
Antagonistas de cálcio	20	26	0,89
Antiplaquetários	53	85	0,013
ECG normal (%)	33,3	46,7	0,51

DMC-DC: doença microvascular coronariana - etiologia chagásica; DMC-OE: doença microvascular coronariana - outras etiologias. IMC: índice de massa corporal; IECA: inibidores da enzima conversora de angiotensina; BRA: bloqueadores dos receptores da angiotensina; ECG: eletrocardiograma

Anormalidades no ECG foram frequentes no grupo DMC-DC, com apenas 33,3% dos pacientes apresentando um ECG normal na data do cateterismo cardíaco. Por outro lado, o grupo DMC-OE não apresentou significativamente mais pacientes com um ECG normal (46,7%). Enquanto o bloqueio de ramo direito (26,6%), o hemibloqueio anterior esquerdo (13,3%) e a sobrecarga do VE (13,3%) foram as anormalidades mais frequentes no grupo DMC-DC, a sobrecarga do VE (20%) e o bloqueio de ramo esquerdo completo (6,7%) foram anormalidades predominantes no grupo DMC-OE. Ambos os grupos tiveram uma prevalência semelhante de fibrilação atrial (6,7%). Nenhuma dessas diferenças entre os grupos foi estatisticamente significante.

### Avaliação Hemodinâmica e Ventriculografia com Contraste

A disfunção diastólica, sugerida pelo aumento da PTDVE > 12 mmHg em repouso, foi diagnosticada em 13 (86,6%) e em 64 (74,4%) dos pacientes, nos grupos DMC-DC e DMC-OE, respectivamente; p=0,511. Os valores médios de PTDVE foram similares em ambos os grupos. Além disso, 9 (60%) e 45 (52,3%) pacientes apresentaram PTDVE > 20 mmHg nos grupos DCM-DC e DCM-OE, respectivamente. Tabela 2 .

**Tabela 2:** – Avaliação hemodinâmica, angiográfica e da perfusão miocárdica nos grupos de pacientes com doença microvascular coronariana associada à cardiomiopatia da doença de Chagas versus DMC por outras etiologias

	DMC-CE n = 15	DMC-OE n = 86	p
Hipertrofia do VE (%)	20,0	53,3	0,128
Dilatação do VE (%)	26,0	4,7	0,04
PTDVE (mmHg)	20,13 ± 5,43	19,0 ± 5,1	0,44
FEVE	54,8 ± 15,9	61,1 ± 11,9	0,049
Alteração da mobilidade do VE (%)	86,6%	53,3%	0,02
EMPVE	1,77 ± 0,35	1,18 ± 0,26	0,01
Defeitos perfusão isquêmica (%)	45,5	62,3	0,31
SDS	0 (0 - 8)	3 (0 - 19)	0,23

DMC-DC: doença microvascular coronariana - etiologia chagásica; DMC-OE: doença microvascular coronariana - outras etiologias; VE: ventrículo esquerdo; PTDVE: pressão telediastólica do ventrículo esquerdo; FEVE: fração de ejeção do ventrículo esquerdo; EMPVE: escore de mobilidade parietal ventricular esquerda; SDS: somatória do escore diferencial (summed differential score). Defeitos perfusionais isquêmicos e seus corrspondentes escores diferenciais foram avaliados em 11 e 8 pacientes nos grupos DMC-DC e DMC-OE, respectivamente

A ventriculografia revelou características morfológicas do VE sugestivas de hipertrofia em três pacientes (20%) do grupo DMC-DC versus 26 pacientes (30,2%) do grupo DMC-OE (p=0,545). Em contraste, dilatação ventricular foi observada em proporção significativamente maior de pacientes do grupo DMC-DC (26%, n=4) em comparação ao grupo DMC-OE (4,7%, n=4) (p=0,04).

**Figura 1:**
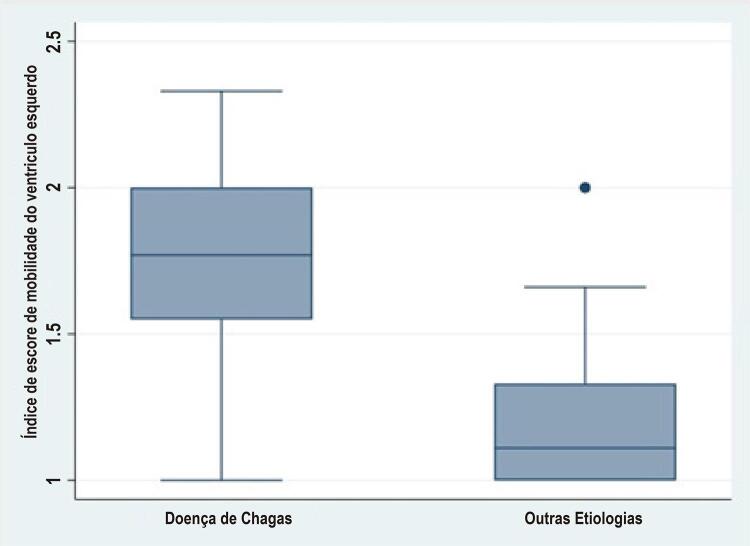
– Escore de mobilidade parietal ventricular esquerda de acordo com a etiologia da doença microvascular coronariana

**Figura 2:**
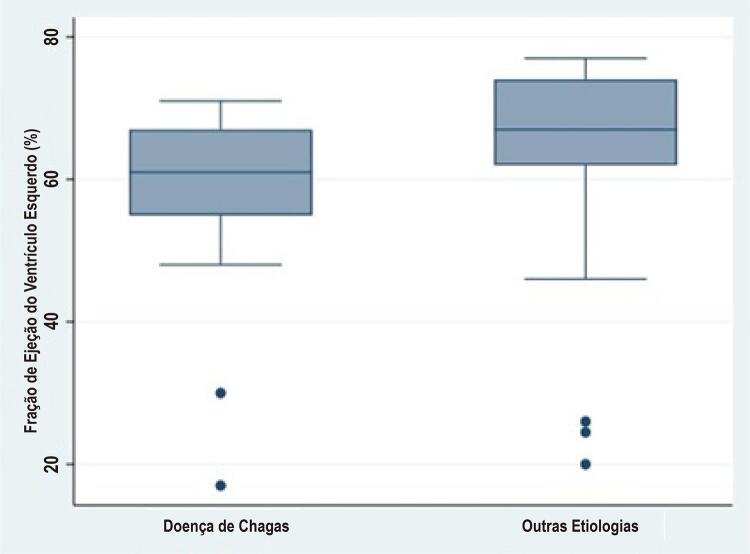
– Comparação da fração de ejeção do ventrículo esquerdo entre os grupos de pacientes com doença microvascular coronariana

No geral, a função sistólica global do VE estava preservada na maioria dos pacientes de ambos os grupos, com uma minoria de pacientes apresentando redução na FEVE (<50%). Observou-se diferença marginalmente significativa entre os valores médios de FEVE do grupo DCM-DC (54,8 ± 15,9) *vs* . DMC-OE (61,1 ± 11,9), p = 0,049 ( Figura 1 ). Adicionalmente, anormalidades na mobilidade parietal ventricular foram detectadas em uma proporção significantemente maior de pacientes com DCM-DC (86,6%) em comparação a de pacientes com CMD-OE (52,2%), p = 0,02 ( Figura 2 ). Finalmente, o escore de movimentação parietal do VE, que computa a extensão e a gravidade de anormalidades da mobilidade segmentar parietal do VE na sístole, foi mais elevado no grupo com DCM-DC (1,77 ± 0,35) que no grupo com DCM-OE (1,18 ± 0,26), p = 0,01.

Avaliação da Isquemia Miocárdica por Cintilografia de Perfusão Miocárdica

Após a obtenção dos resultados da avaliação hemodinâmica, 11 pacientes do grupo DCM-DC e oito pacientes do grupo DCM-OE foram submetidos à avaliação funcional com cintilografia de perfusão miocárdica SPECT ( Tabela 2 ).

As proporções de pacientes exibindo anormalidades isquêmicas reversíveis na perfusão nos grupos DCM-DC e DCM-OE foram 45,5% e 62,3%, respectivamente (p = 0,31). O SDS também não foi diferente entre os grupos DCM-DC (1,91 ± 3,05) e DCM-OE (5,63 ± 7,03) (p = 0,134).

## Discussão

Durante o ano de 2018, após aplicar os critérios de inclusão e exclusão a uma série de 247 pacientes consecutivos clinicamente estáveis, que não apresentavam doença cardíaca estrutural e queixaram-se de sintomas anginosos graves o suficiente para justificar o encaminhamento ao nosso centro terciário de coronariografia invasiva (que evidenciou artérias coronárias epicárdicas normais), uma amostra considerável de 101 pacientes (40,9%) concordou em participar deste estudo prospectivo. Dos 101, cerca de um sexto (15 pacientes) apresentou resultado positivo para infecção crônica pelo *T. cruzi* e compôs o grupo com provável doença microvascular coronariana causada por CCDC (DMC-DC), enquanto os outros 86 pacientes compuseram o grupo com provável doença microvascular coronariana cuja causa foi atribuída a outras etiologias (DCM-OE). Este é o primeiro relato sobre a prevalência relativa de CCDC como etiologia de disfunção microvascular entre pacientes que geralmente são considerados como portadores de uma forma primária de doença microvascular coronariana. É provável que esse número de aproximadamente 15% corresponda à estimativa real dessa etiologia, considerando que nossa instituição ainda recebe muitos pacientes de regiões endêmicas da DC. Além disso, nossa amostra de 247 pacientes com artérias coronárias angiograficamente normais foi selecionada entre 691 pacientes consecutivos, nos quais outras anormalidades foram excluídas, e corresponde a quase 36% dos indivíduos encaminhados para angiografia coronariana eletiva no período. Esse resultado é inferior aos 45% relatado recentemente por outro hospital brasileiro,^25^ mas é muito semelhante aos 39% de pacientes com estenoses coronárias < 20% submetidos à angiografia coronária eletiva entre os anos de 2004 e 2008, segundo o registro nacional do *American College of Cardiology* .^26^

Com exceção da média de idade discretamente mais elevada no grupo DMC-OE, vale ressaltar que os dois grupos em nosso estudo apresentavam características antropométricas e clínicas essencialmente similares, incluindo uma maior prevalência do gênero feminino, pacientes ligeiramente obesos que, além da angina atípica, também se queixaram de dispneia e palpitações. Os fatores de risco tradicionais para doença arterial coronariana - hipertensão, *diabetes mellitus,* tabagismo, dislipidemia - também estavam presentes em proporções comparativamente altas de pacientes em ambos os grupos.

Outro resultado importante do nosso estudo é que, usando a ventriculografia por contraste radiológico para avaliação morfológica do ventrículo esquerdo, foram encontrados sinais de hipertrofia em vários pacientes de ambos os grupos. Em concordância com esse resultado, a disfunção diastólica do VE, expressa por conspícua elevação da pressão telediastólica ventricular, ocorreu de forma comparável nos grupos DMC-DC e DMC-OE.

Nossos pacientes com DMC por DC, considerando a fase da doença em que se encontravam quando recrutados para esse estudo, exibem anormalidades que, segundo classificação recente de DMC, manifestam-se principalmente na insuficiência cardíaca com FEVE preservada.^27^ Embora a disfunção diastólica também tenha ocorrido no grupo com DMC decorrente de outras etiologias, é provável que a fibrose miocárdica, uma característica marcante da CCDC, contribua para a disfunção diastólica aqui detectada em nossos pacientes.^6^

Contudo, houve ainda diferenças relevantes entre os dois grupos em relação à função sistólica do VE. A FEVE média foi menor no grupo DMC-DC, condizente com o escore mais elevado de mobilidade segmentar parietal ventricular mais deteriorada e com maior dilatação da câmara, significativamente mais prevalente no grupo DMC-DC comparado ao grupo DMC-OE.

Ambos os grupos exibiram proporções similares de pacientes com defeitos de perfusão e os escores SDS entre cintilografia miocárdica com estresse e em repouso também foram semelhantes. Importante enfatizar que a detecção de anormalidades da perfusão miocárdica, indicativas de isquemia miocárdica induzida pelo estresse, foi o terceiro critério para classificar nossos pacientes como portadores da provável síndrome da doença microvascular coronariana, segundo a mais recente classificação padronizada.^28^ Embora nessa padronização seja sugerido que um quarto critério possa ser usado quando houver suspeita de angina microvascular, na presente investigação não se aplicou nenhum teste adicional para certificar a ocorrência de comprometimento da função microvascular coronariana, como o cálculo de índices de resistência ou de redução da reserva de fluxo, pois esse aspecto escapava ao contexto da pesquisa.^28^ No entanto, é provável que alguns de nossos pacientes do grupo DMC-DC exibam tais anormalidades, conforme relatado por outros pesquisadores.^29^

É razoável supor que os pacientes de ambos os grupos incluídos nesta pesquisa compartilham características fisiopatológicas comuns envolvidas no aparecimento de angina em indivíduos com artérias subepicárdicas angiograficamente normais, implicando a presença de distúrbios no nível microvascular coronariano. Esse conceito é sustentado pela identificação de fatores como hipertensão arterial, hipertrofia ventricular e disfunção diastólica em ambos os grupos. Entretanto, no grupo com DMC associada à infecção crônica por *T. cruzi* , é provável que as peculiaridades inerentes à CCDC tenham sido responsáveis pelas manifestações relativamente mais graves da disfunção sistólica do ventrículo esquerdo, em comparação com as exibidas pelo grupo com DMC por outras etiologias.

É importante salientar que nenhum dos pacientes que testou positivo para anticorpos contra o *T. cruzi* em nosso estudo possuía conhecimento prévio sobre o acometimento pela doença de Chagas. Além disso, a amostra de pacientes selecionados para participar do estudo foi composta primariamente por pessoas encaminhadas para angiografia coronária invasiva, sem avaliação prévia de isquemia miocárdica com exames como o teste ergométrico com ECG, ecocardiografia sob estresse, ou cintilografia miocárdica nuclear. Assim, é provável que a maioria desses pacientes possa se beneficiar de medidas terapêuticas direcionadas tanto pelo conhecimento de sua doença de base quanto pelas consequências anatômicas e funcionais dos distúrbios microvasculares.^30^

### Limitações

Não foram realizadas investigações específicas para determinar a possível etiologia da DMC nos pacientes sem CCDC, que muito provavelmente viriam a ser classificados como portadores de DMC primária. Além disso, não foram realizados testes invasivos para explorar diretamente os mecanismos responsáveis pelo comprometimento da função microvascular nos pacientes do estudo. Outra limitação reside no fato de que apenas 19 dos 101 pacientes incluídos foram avaliados com cintilografia miocárdica de perfusão SPECT. Dessa forma, embora proporções semelhantes de pacientes com distúrbios de perfusão tenham sido encontradas entre os grupos, o pequeno número de pacientes pode ter dificultado a detecção de diferenças significativas entre eles.

## Conclusão

A CCDC foi etiologicamente associada à DMC em um sexto da amostra de 101 pacientes consecutivos estáveis, cujo sintoma principal era dor anginosa, requerendo elucidação por angiografia coronária invasiva. Esses pacientes infectados cronicamente com *T. cruzi* apresentaram características antropométricas, clínicas e angiográficas, bem como anormalidades hemodinâmicas e de perfusão miocárdica, semelhantes às detectadas nos 86 pacientes com outras etiologias para a provável disfunção microvascular. No entanto, o comprometimento da função sistólica segmentar e global do VE foi significativamente mais grave nos pacientes com sintomas de disfunção microvascular relacionada à CCDC.
