# Supplementary material for: Estudo Comparativo da Doença Coronariana Microvascular Causada por Doença de Chagas e por Outras Etiologias
Source: Arq Bras Cardiol. 2020 Dec 1;115(6):1094–101. [Article in Portuguese] doi: 10.36660/abc.20200381 (PMC8133735; doi:10.36660/abc.20200381)
Supplement: Translation

# Translation: Chagas Cardiomyopathy as the Etiology of Suspected Coronary Microvascular Disease. A Comparison Study with Suspected Coronary Microvascular Disease of Other Etiologies

**Authors:** Felipe Araujo Campos, Mariana L. Magalhães, Henrique Turin Moreira, Rafael B. Pavão, Moyses O. Lima-Filho, Igor M. Lago, André V. Badran, João R. A. Chierice, André Schmidt, José Antonio Marin-Neto

**Affiliations:** 1 Brazil Universidade de São Paulo Faculdade de Medicina de Ribeirao Preto, Ribeirão Preto, SP - Brazil

**Keywords:** Chagas Cardiomyopathy, Coronary Microvascular, Dysfunction, Dysfunction Ventricular, Left, Diastolic Dysfunction, Wall Motion Abnormality Index, Left Ventricular Ejection Fraction

## Abstract

**Background:**

Chagas disease (CD) as neglected secondary form of suspected coronary microvascular dysfunction (CMD).

**Objectives:**

Comparison of patients with CMD related to CD (CMD-CE) versus patients with CMD caused by other etiologies (CMD-OE).

**Methods:**

Of 1292 stable patients referred for invasive coronary angiography to elucidate the hemodynamic pattern and the cause of angina as a cardinal symptom in their medical history, 247 presented normal epicardial coronary arteries and 101 were included after strict exclusion criteria. Of those, 15 had suspected CMD-CE, and their clinical, hemodynamic, angiographic and scintigraphic characteristics were compared to those of the other 86 patients with suspected CDM-OE. Level of significance for all comparisons was p < 0.05.

**Results:**

Patients with suspected CMD-CE showed most anthropometric, clinical, angiographic hemodynamic and myocardial perfusion abnormalities that were statistically similar to those detected in the remaining 86 patients with suspected CMD-OE. LV diastolic dysfunction, expressed by elevated LV end-diastolic pressure was equally found in both groups. However, as compared to the group of CMD-OE the group with CMD-CE exhibited lower left ventricular ejection fraction (54.8 ± 15.9 vs 61.1 ± 11.9, p= 0.049) and a more severely impaired index of regional wall motion abnormalities (1.77 ± 0.35 vs 1.18 ± 0.26, p= 0.02) respectively for the CMD-OE and CMD-CE groups.

**Conclusion:**

Chronic Chagas cardiomyopathy was a secondary cause of suspected coronary microvascular disease in 15% of 101 stable patients whose cardinal symptom was anginal pain warranting coronary angiography. Although sharing several clinical, hemodynamic, and myocardial perfusion characteristics with patients whose suspected CMD was due to other etiologies, impairment of LV segmental and global systolic function was significantly more severe in the patients with suspected CMD related to Chagas cardiomyopathy. (Arq Bras Cardiol. 2020; 115(6):1094-1101)

## Introduction

More than one century after its discovery in 1909 Chagas disease (CD) is still a major public health problem in Latin America and, due to migratory moves during the last decades, also in non-endemic areas, such as the United States and some European countries.^1 , 2^Chronic Chagas cardiomyopathy (CCC) is the most prevalent and more ominous of the clinical manifestations of CD, being essentially due to a low-grade but virtually incessant form of infectious myocarditis that is characteristically diffuse but with focal myocitolytic necrosis and intense reparative fibrosis.^3 , 4^Since in experimental models of ischemia/reperfusion myocitolytic necrosis is usually associated with low intensity but repetitive hypoxic or ischemic damage, this type of myocardial lesion has been interpreted as a consequence of microvascular ischemic disturbances and constitute one of the four main pathogenetic mechanisms that lead to CCC.^5 , 6^

The occurrence of microvascular ischemia in patients chronically infected with the *T. cruzi* has been demonstrated by various pathological, clinical and experimental laboratory evidence.^7 - 9^An estimated 20-40% of patients with CCC complain of angina, usually atypical in character since the symptom has no constant relation to physical or emotional stimulation, and variable duration and response to nitrates.^10^Also, several independent investigators have shown that these patients have striking myocardial perfusion abnormalities that are elicited by exercise and reversible with rest, in the presence of coronary arteries that have no epicardial obstructive lesions at invasive angiography^11 - 13^These perfusion abnormalities are therefore attributable to microvascular ischemia and are corroborated by studies in experimental models of *T. cruzi* infection.^14 , 15^

On the basis of such evidence, many patients with CCC could now be classified as having a secondary class of coronary microvascular disease (CMD), related to a cardiomyopathic process caused by an inflammatory infectious disease.^16 - 18^

There have been no specific studies focusing on the comparison of patients with CMD related to CD versus patients with CMD linked to other etiologies. Thus this was the objective of the present investigation.

## Methods

### General Design of the Study and Sample Population

This was a transversal, observational, unicentric investigation with prospective inclusion of stable patients who were referred to our tertiary academic hospital from January 01 to December 31 of 2018 for invasive contrast coronary angiography to elucidate the hemodynamic pattern and the cause of angina as a cardinal symptom in their medical history. Of 1292 such patients 601 were excluded because of: previous treatment with coronary angioplasty (200); previous confirmed acute coronary syndrome (137); valvular heart disease (113); hypertrophic or idiopathic dilated cardiomyopathy (99); previous coronary artery bypass surgery (49) and cardiac transplantation (3).

In the remaining 691 patients coronary angiography indicated to specifically evaluate the primary possibility of coronary artery disease, revealed: significant obstructive epicardial lesions (stenoses > 40% of luminal diameter reduction): (n = 367); angiographycally normal coronary arteries: (n = 247); non-significant epicardial coronary artery disease - stenosis < 40%: (n = 77); miscellaneous coronary artery conditions - congenital abnormalities, myocardial bridge, excessive tortuosity or ectasia, coronary-cavitary fistula, slow flow: (n = 81).

Of the 247 patients who were clinically stable, with no structural cardiac disease and whose cardinal symptom was angina severe enough to warrant the indication of invasive coronary angiography but that did not show any abnormalities, 101 agreed to participate in the study and signed the informed consent. The research protocol was approved by the Ethics Committee of the Hospital das Clínicas da Faculdade de Medicina de Ribeirão Preto - USP - Processes : 8430/2011 and CAEE 07494618.3.0000.5440 Doc 3.252.539.

### Clinical and Laboratory Assessment

The 101 patients enrolled into the study had a clinical assessment performed in the same day as the hemodynamic and the invasive coronary evaluation, comprising the characterization of angina, the presence of associated symptoms such as dyspnea, fatigue, edema, palpitation, syncope, disfagia, intestinal constipation, and epidemiological hints of having been exposed to the *T. cruzi* infection. Also included was a questionnaire regarding the occurrence of risk factors for coronary artery disease, such as: systemic hypertension, diabetes mellitus, dyslipidemia, current smoking, and familial history of early coronary disease. All patients had a 12-lead ECG recorded, and a sub-sample of 33 (9 and 24 in each group) was examined with a transthoracic rest echocardiogram. All 101 patients had a serological examination for the detection of circulating antibodies against the *T. cruzi* . Also, a peripheral blood sample was collected from all patients, to allow the examination of serum levels of creatinine, and exclude the presence of renal and liver damage, as well as of severe anemia and diabetes mellitus.

### Cardiac Catheterization, Hemodynamic Assessment and Coronary Angiography

These procedures were performed under local anesthesia preferably using the radial approach with conventional guide-wires and catheters. Manual injections of 3-7ml of radiological contrast were selectively done in each coronary ostium,^19^with recording at 15-30 frames/sec, in several projections. Left ventricular end-diastolic pressure (LVEDP) was recorded at rest, followed by two automatic injections of 20-30ml of dye at 8-10ml/sec and recording at 15 frames/sec, in the right and left oblique projections. The contrast ventriculography was then analysed according to a 9-segment model using a quantitation score that ascribed 1 to normal wall motion, 2 to hypokinesis, 3 to akinesia and 4 to dyskinesis.^20^An index of the global wall motion was obtained by summing the segmental scores divided by the number of segments analysed.^21^The ventriculogram also allowed the qualitative assessment of LV hypertrophy and dilation.

### SPECT Myocardial Scintigraphy

A subset of 19 patients were submitted to rest-exercise SPECT myocardial scintigraphy. The images were acquired in the camera range (Philips BrightView XCT - Cleveland, OH) of a double detector with the patient in the supine position during rest and stress phases. The acquisition occurred in semicircular orbit (180 degrees, from right anterior oblique projection to the left posterior oblique projection), in 32 projections synchronized with the electrocardiogram, 8 frames per cardiac cycle in 60 seconds by projection (“accepted” heartbeat) with 50% acceptance window around of average R-R. The detectors were equipped with collimators of parallel holes of low energy and high resolution, using a 64 x 64 pixel acquisition matrix.^22^

Physical exercise was used as the preferred stress test, beta-blockers, calcium channel blockers and other anti-ischemic drugs were interrupted 48 hours before the nuclear tests. Sestamibi-Tc99m was used as a radiotracer to assess regional myocardial perfusion, at a dose of 12 to 15 mCi at rest and 25 to 30 mCi at stress. Images were acquired 1 hour after each intravenous injection of the radiotracer.

Polar maps using the 17-segment model were generated to assess the perfusion abnormality according to a score defined as 0= normal, 1= slight uptake reduction, 2= moderate uptake reduction, 3= marked uptake reduction, and 4= no tracer uptake. Perfusion abnormalities in stress (SSS- Summed Stress Score) and rest (SRS- Summed Rest Score) were quantified to differentiate between reversible, when Summed Difference Score (SDS) ≥ 1 and irreversible perfusion defects.^23^

### Statistical Analysis

On the basis of the specific serology tests for diagnosing antibodies against the *T. cruzi* , 15 patients were classified as having suspected CMD due to Chagas etiology (CMD-CE) - two positive serological tests with different methods.^24^The other 86 patients, with negative serology tests, composed the group of patients with suspected CMD caused by other etiologies (CMD-OE). The Shapiro-Wilk tests were used to check if variables had a normal distribution, in which unpaired variables were compared with Student’s “t” tests, otherwise the Mann-Whitney unpaired tests were used. Continuous variables with normal distribution were described as mean ± standard deviation while non-normal distributed variables were described as median and IQ or range interval. Categorical variables were described as absolute or relative values (percentages or proportions). Proportions within each group were compared using Fisher exact tests. All tests were bicaudal, with p< 0.05 considered significant. All analyses were done with the Stata software *(StataCorp, EUA, versão 14.2)* .

## Results

### Clinical Features - (Table 1)

Of the 247 stable patients who were initially eligible since they presented no exclusion criteria to enter the study and fulfilled the inclusion criteria of having epicardial coronary arteries that were normal at angiography, 101 (40.9%) were actually enrolled. Of those, only 15 (14,8%) had two serologic tests positive for *T. cruzi* , and composed the group CMD-CE with 40% of males and average age of 61.3 ± 6.7 years). The other 86 (85.2%) composed the group CMD-OE with 32.5% men and higher mean age of 68.9 ± 11.0 years.

On the date of entry into the study, atypical angina was referred by 9 (60%) of the group CMD-CE versus 57 (66%) patients of the group CMD-OE, with typical angina referred by the other patients of each group. In group CMD-CE dyspnea and palpitation, were also frequent, with 8 (53%) and 7(47%) patients respectively, versus 55 (64%) with dyspnea and only 30 (35%) with palpitation in group CMD-OE.

Systemic arterial hypertension was the most prevalent risk factor for coronary artery disease in both groups, with 93.3% vs 81.3%, followed by diabetes mellitus with 40.0% vs 33.7%, dyslipidemia with 33.3 vs 41.8% and active smoking in 13.3 vs 24.4% respectively in the CMD-CE and CMD-OE groups. Both groups were using statistically similar proportions of pharmacological drugs against hypertension, diabetes mellitus, statins and agents to control myocardial ischemia symptoms, such as antiplatelets and calcium antagonists. ( Table 1 ). However, ACE-inhibtors/ARBs were more used by the CMD-CE group while antiplatelet agents were more used by the CME-OE patients.

**Table 1:** – Demographic and baseline clinical characteristics of patients enrolled according to the etiology of coronary microvascular disease

	CMD-CE n = 15	CMD-OE n = 86	p-value
Age (years)	61.3 ± 6.7	68.9 ± 11.0	0.01
Female Gender (%)	60.0	67.4	0.65
Body weight (kg)	77.0 ± 13.1	80.7 ± 15.3	0.18
BMI (kg/m^2^)	31.0 ± 7.3	31.9 ± 5.6	0.72
Atypical angina (%)	60.0	66.3	0.86
Dyspnea (%)	53.0	64.0	0.62
Palpitation (%)	47.1	35.2	0.56
Hypertension (%)	93.3	81.3	0.46
Diabetes mellitus (%)	40	33.7	0.77
Dyslipidemia (%)	33	53.5	0.58
Smoking (%)	13.3	24.4	0.51
**Use of medications**			
ACEI/ARB	100	71	0.037
Beta-blockers	53	54	0.79
Statins	47	53	0.90
Antidiabetics	40	42	0.88
Diuretics	47	40	0.82
Nitrates	20	12	0.63
Calcium antagonists	20	26	0.89
Antiplatelets	53	85	0.013
Normal EKG (%)	33.3	46.7	0.51

*CMD-CE: coronary microvascular disease - Chagas etiology; CMD-OE: coronary microvascular disease - other etiologies. BMI: body mass index; ACEI: angiotensin converter enzyme inhibtors; ARB: angiotensin receptor blockers; EKG: electrocardiogram.*

ECG abnormalities were frequent in the group CMD-CE, with only 33.3% of patients having a normal ECG on the date of the catheterization. In contrast, the group of CMD-OE had non-significantly more patients with a normal ECG (46.7%). While RBBB (26.6%), LAHB (13.3%) and LV overload (13.3%) were the most frequent abnormalities in the CMD-CE group, LV overload (20%) and complete LBBB (6.7%) were predominant abnormalities in the CMD-OE group. Both groups had a similar prevalence of atrial fibrillation (6.7%). None of those differences was statistically significant.

### Hemodynamic and Contrast Ventriculography Assessment. (Table 2).

Diastolic dysfunction as suggested by increased LVEDP > 12 mmHg at rest was diagnosed in 13 (86.6%) and 64 (74.4%) of patients respectively in the groups CMD-CE and CMD-OE, respectively; p= 0.511. The mean values of LVEDP were similar in the groups. In addition, 9 (60%) and 45 (52.3%) patients had LVEDP > 20mmHg respectively in the CMD-CE and CMD-OE groups.

Using ventriculography to assess LV morphological features that suggest the presence of chamber hypertrophy this alteration was observed in 3 patients (20%) of the group CMD-CE versus 26 patients (30.2%) of the group CMD-OE (p= 0.545). In contrast ventricular dilatation by ventriculography was observed in significantly higher proportion of patients, 26% (n = 4) of the group CMD-CE versus 4.7% (n = 4) of the group CMD-OE (p= 0.04).

Overall most patients had preserved global LV systolic function in both groups, with a minority of patients in both groups showing reduced LVEF values (< 50%). LVEF was marginally significant lower in the CMD-CE (54.8 ± 15.9) as compared to the CMD-OE group (61.1 ± 11.9 ), p= 0.049 (Figura 1). In addition, regional wall motion abnormalities were detected in a significantly higher proportion of patients in the CMD-CE group (86.6%; n = 13) as compared with that of patients in the CMD-OE group (52.2%; n = 45), p= 0.02 ( Figure 2 ). LV wall motion score index that computs the extent and severity of the segmental systolic abnormality was also higher in the CMD-CE, of 1.77 ± 0.35 than in the CMD-OE group of 1.18 ± 0.26; p= 0.01.

**Figure 2:** – Comparison of Left Ventricular Ejection Fraction in both groups of patients with coronary microvascular dysfunction.

### Assessment of Myocardial ischemia with myocardial perfusion scintigraphy

After the results of the hemodynamic evaluation were obtained, 11 patients of the CMD-CE group and 8 patients of the CMD-OE group underwent functional assessment with SPECT-myocardial perfusion scintigraphy. ( Table 2 ).

**Table 2:** – Hemodynamic, angiographic and myocardial perfusion evaluation in the groups of patients with coronary microvascular dysfunction associated to Chagas cardiomyopathy versus CMD due to other etiologies

	CMD-CE n = 15	CMD-OE n = 86	p
LV hypertrophy (%)	20	53.3	0,128
LV dilation (%)	26.0	4.7	0.04
LVEDP (mmHg)	20.13 ± 5.43	19.0 ± 5.1	0.44
LVEF	54.8 ± 15.9	61.1 ± 11.9	0.049
LV segmental abnormalities (%)	86.6%	53.3%	0.02
LVWMSI	1.77 ± 0.35	1.18 ± 0.26	0.01
Ischemic perfusion defects (%)	45.5	62.3	0.31
SDS	0 (0 - 8)	3 (0 - 19)	0.23

*CMD-CE: coronary microvascular disease - Chagas etiology; CMD-OE: coronary microvascular disease - other etiologies; LV: left ventricle; LVEDP: left ventricular end-diastolic pressure; LVEF: left ventricular ejection fraction; LVWMSI: left ventricular wall motion score index; SDS: summed difference score. Ischemic perfusion defects, with their correspondent SDS values were evaluated in 11 and 8 patients respectively of the CMD-CE and CMD-OE groups.*

The proportion of patients exhibiting ischemic reversible perfusion abnormalities for the CMD-CE and CMD-OE groups were 45.5% and 62.3% respectively (p= 0.31). The SDS was also similar for the CMD-CE (1.91 ± 3.05) and the CMD-OE (5.63 ± 7.03) groups (p= 0.134).

## Discussion

During the whole year of 2018, following the implementation of our inclusion and exclusion criteria to a consecutive series of 247 clinically stable patients who had no structural cardiac disease and complained of anginal symptoms that were severe enough to warrant referral to our tertiary center for invasive coronary angiography but that eventually showed normal epicardial coronary arteries, a sizable sample of 101 of them (40.9%) agreed to participate in this prospective study and signed the respective informed consent. Out of the 101 about one sixth tested positive for chronic *T. cruzi* infection, and composed the group of patients with suspected coronary microvascular disease due to Chagas cardiomyopathy etiology (CMD-CE), while the other 86 patients comprised the group whose suspected coronary microvascular disease must be ascribed to other etiologies (CMD-OE). This is the first report on the relative prevalence of Chagas cardiomyopathy as an etiology for suspected coronary microvascular dysfunction among patients who are otherwise generally considered as having a primary form of coronary microvascular disease. This figure of roughly 15% of is likely to correspond to the real estimate for that etiology, considering that our institution still receives many patients from regions endemic for Chagas disease. Also, our sample of 247 patients with angiographically normal coronary arteries was selected among 691 consecutive patients in whom other abnormalities had been excluded, and correspond to nearly 36% of such individuals who are referred for elective coronary angiography. This finding is inferior to 45% recently reported by another Brazilian hospital,^25^but is quite similar to the reported 39% of patients with coronary stenoses < 20% as the general yield by elective coronary angiography during the years 2004-2008 from an American College of Cardiology national registry.^26^

With the exception of slightly higher mean age in the CMD-OE group, it is noteworthy that the two groups in our study had essentially similar anthropometric and clinical characteristics, including a higher prevalence of female gender, with slighthly obese patients who, in addition to atypical angina, also complained of dyspnea and palpitation. The traditional risk factors for coronary artery disease - hypertension, diabetes mellitus, smoking, dyslipidemia - were also present in comparably high proportions of patients of both groups.

Another important finding from our study is that using contrast ventriculography for evaluation of left ventricle morphology, signs of chamber hypertrophy were found in several patients from both groups. In line with these findings, LV diastolic dysfunction, as expressed by striking elevation of the LV end-diastolic pressure occurred similarly in the CMD-CE and CMD-OE groups.

In regard to this important derangement, our patients with CMD due to Chagas disease at the stage of the disease they are when enrolled in this study, show abnormalities that are described in the recent classification of coronary microvascular dysfunction as occurring mostly in the scenario of heart failure with preserved LV ejection fraction.^27^Although diastolic dysfunction also occurred in the group with CMD due to other etiologies, it is likely that myocardial fibrosis, a hallmark of Chagas cardiomyopathy, contributes to the diastolic dysfunction hereby detected in our patients.^6^

However, there were relevant differences between the two groups regarding LV systolic function. Mean LV ejection fraction was significantly lower in the CMD-CE, in keeping with more deteriorated segmental wall motion index and more LV dilation that was significantly more prevalent in the CMD-CE as compared to the CMD-OE group.

Both groups exhibited comparable proportions of patients with ischemic perfusion defects and their SDS between stress and rest myocardial scintigraphy were also similar. It is worthy pointing out that the objective detection of myocardial perfusion abnormalities indicative of myocardial ischemia induced by stress, represents the third criteria for classifying the patients in our study series as having the suspected syndrome of coronary microvascular disease according to a standardized recent classification.^28^Although in this standardization it is suggested that a fourth criterium could be used when the presence of microvascular angina is suspected, in our investigation we did not apply any additional tests to certify the occurrence of impaired coronary microvascular function, such as measurement of resistance indices or of reduced flow reserve.^28^However it is probable that at least some of our patients with CMD-CE could have such abnormalities, as reported by other investigators.^29^

It is reasonable to assume that patients from both groups included in this research share common pathophysiological characteristics that are involved in the appearance of the syndrome of anginal pain with angiographically normal sub-epicardial arteries, thus implying the presence of disturbances at the coronary microvascular level. This concept is supported by the finding in both groups of factors such as hypertension, ventricular hypertrophy, and diastolic dysfunction. However, in the group whose coronary microvascular disease is associated with the chronic *T. cruzi* infection, it is likely that the inherent peculiarities of Chagas cardiomyopathy be responsible for the relatively more serious manifestations of left ventricular systolic dysfunction, in comparison with those exhibited by the group with coronary microvascular disease due to other etiologies.

It is relevant to emphasize that none of the patients who tested positive for antibodies against the *T. cruzi* in our study had previous knowledge about harboring Chagas disease. Moreover, the sample of patients eventually selected to participate in the study was composed primarily of people who were referred to invasive coronary angiography, without previous assessment of myocardial ischemia with exams such as ECG-based tests, or stress echocardiography or nuclear scintigraphy tests. Thus, it is likely that most of those patients could benefit from therapeutic measures directed by both the knowledge of their baseline disease and of the functional consequences of the microvascular disturbances.^30^

### Limitations

No specific investigations were carried out to determine the possible etiology in the patients without Chagas cardiomyopathy, although most probably they would be classified as having primary coronary microvascular dysfunction. Also, no invasive tests were done to directly explore the mechanisms responsible for the impairment of microvascular function in any patient included in this study. “Another limitation is the fact that only 19 of the 101 patients had myocardial pefusion assessed with SPECT scintigraphy. Hence, although similar proportions of the patients had perfusion defects, the small number of patients in both groups may have prevented detection of differences in regard to this important characteristic of their coronary microvascular derangements”.

## Conclusions

Chronic Chagas cardiomyopathy was found in association with the suspected syndrome of coronary microvascular disease in one sixth of a sample of 101 stable patients whose cardinal symptom was anginal pain warranting invasive coronary angiography. These patients chronically infected with the *T. cruzi* showed anthropometric, clinical, and angiographic characteristics, as well as hemodynamic and myocardial perfusion abnormalities that were similar to those detected in the remaining 86 patients with other etiologies for the suspected microvascular dysfunction. However, impairment of LV segmental and global function was signficantly more severe in the patients with symptoms of possible microvascular dysfunction related to Chagas cardiomyopathy.

**Figure 1:** – Left Ventricular Wall Motion Score Index according to etiology.
